# Parliament and the Profession

**Published:** 1916-03-25

**Authors:** 


### PARLIAMENT AND THE PROFESSION.

### The Army Medical Service.

Since our last report appeared a sensible proportion of the time of the House of Commons has been devoted to praise and criticism of the Army Medical Service. In his statement in presenting the Army Estimates, Mr. Tennant spoke in the highest terms of all branches of the Medical Service. At Lapugnoy, with its 376 beds, no fewer than 2,000 casualties were dealt with in one day, while at the clearing station at Bailleul the vast number of 61,000 sick and wounded have passed through the hospital since it was opened, while the number of major operations reaches to over 1,000.

### An Attack on the System of Organisation.

On the following day Mr. R. McNeill made a long attack on the medical arrangements of our Armies in the field, the report of his speech occupying twenty columns of "Hansard." He urged that the organisation which was adapted to old-time warfare was quite unsuited for the fighting of to-day. At Loos, he said, thousands of men were left lying in the fields and streets all night because the hospitals were all full and the staffs were overworked, while within a short distance there were hundreds of doctors of other divisions doing nothing. The fault was not with the doctors, but with the organisation. He was told that the field hospitals near the Front served no real purpose whatever. "A distinguished surgeon who crosses over to France on a visit to see how things are going on does not really have the opportunity of testing the system at work. He goes to one particular clearing station or hospital in a certain section of the line and sees things in a perfect condition . . . and comes back and reports accordingly." Mr. McNeill's speech included what was subsequently described as "a very bitter and vicious attack upon two distinguished officers—Sir Arthur Sloggett and Surgeon-General Macpherson." Alleging that the organisation had failed to make the best use possible of the medical talent at the disposal of the nation, he said, "Hon. members may remember the story of the lady who was asked the breed of a terrier belonging to her. She replied, 'I think it is just a dog.' In the same way the War Office apparently looks on medical men as 'just doctors.' It does not appear to matter to it whether a man be a surgeon or a physician, a heart specialist or a lung specialist, a chiropodist or a dentist. They just send him to a job and tell him he is wanted there." Finally, Mr. McNeill suggested that three or four thoroughly competent persons should be asked to go to France and to visit the hospitals there, to examine practitioners who have been in the hospitals here and at the Front, to investigate the whole matter, and to discover whether the impressions formed by distinguished surgeons by occasionally visiting a particular point at the Front really present the true aspect of the general working of the whole Medical Service.

### A Reply to Criticisms.

A vigorous reply to Mr. McNeill's attack was made by Colonel Lee, Military Parliamentary Secretary to the Ministry of Munitions. At the commencement of the war Colonel Lee accepted an appointment as Lord Kitchener's special personal representative to proceed to the Front to watch and report upon the practical working of the Medical Services in the field. As a result of nine months' experience he was utterly unable to recognise in the harrowing tale which Mr. McNeill gave of the conditions of the Medical Services anything which in the faintest degree corresponded to the facts as he (Colonel Lee) saw them. He did not know whether the informants who supplied Mr. McNeill with the material for his fancy attack on Sir Arthur Sloggett and Surgeon-General Macpherson combined "the organising skill of Napoleon with the scientific genius of Lister and Paget"—qualifications which Mr. McNeill suggested should be possessed by the person in Sir Arthur Sloggett's position—but he confessed he had met in this country surgeons who had given him the impression that they probably held a higher view of their qualifications than is held generally by their colleagues and by the profession at large. "I would say this about Sir Arthur Sloggett," continued Colonel Lee; "he has great qualities for the head of the Service in the field. He has great experience, extraordinary tact, and great powers of dealing with the difficult personal situations which are bound to arise, like that which arose at the beginning of the campaign between the Royal Army Medical Corps and the Red Cross." Colonel Lee's opinion of the Royal Army Medical Corps is that "there is no finer corps in the British Army, no branch of the Service which has done finer, more effective, and more gallant service during the present war."

### Other Matters of Interest.

Tuberculous Soldiers.—Of eighty-seven cases of men discharged in January 1916 for tuberculosis contracted in France, seventy were granted pensions at war rates, nine have been granted final pensions, and eight cases are still under consideration. In making a statement to this effect Mr. Tennant repudiated the suggestion that hundreds of these cases had now come back on to the Insurance Act.

Discipline in V.A.D. Hospitals.—Sir Clement Kinloch-Cooke asked whether complaints had been received from V.A.D. hospitals as to the strict nature of the regulations governing the movements of convalescent wounded. Mr. Tennant replied that complaints had been received that the discipline was too lax, and also, in smaller numbers, that the discipline was too severe. New instructions having been issued, the complaints ceased.

